# Gut Dysbiosis and Cardiovascular Health: A Comprehensive Review of Mechanisms and Therapeutic Potential

**DOI:** 10.7759/cureus.67010

**Published:** 2024-08-16

**Authors:** Shubam Trehan, Gurjot Singh, Gaurav Bector, Prateek Jain, Tejal Mehta, Kanishka Goswami, Avantika Chawla, Aayush Jain, Piyush Puri, Nadish Garg

**Affiliations:** 1 Internal Medicine, Dayanand Medical College and Hospital, Ludhiana, IND; 2 Internal Medicine, Maharaj Sawan Singh Charitable Hospital, Beas, IND; 3 Internal Medicine, Icahn School of Medicine at Mount Sinai, Queens Hospital Center, New York, USA; 4 Division of Cardiology, Memorial Hermann Pearland Hospital, Pearland, USA; 5 Division of Cardiology, Memorial Hermann Southeast Hospital, Houston, USA

**Keywords:** microbial metabolites, metabolic pathways, molecular mechanisms, cardiovascular health, gut microbiota

## Abstract

Cardiovascular diseases (CVDs) are a leading cause of mortality worldwide. Recent research has identified gut dysbiosis - an imbalance in the gut microbiota - as a significant factor in the development of CVDs. This complex relationship between gut microbiota and cardiovascular health involves various mechanisms, including the production of metabolites such as trimethylamine N-oxide (TMAO) and short-chain fatty acids (SCFAs). These metabolites influence lipid metabolism, inflammation, and blood pressure regulation. In addition, the gut-brain axis and neurohormonal pathways play crucial roles in cardiovascular function. Epidemiological studies have linked gut dysbiosis to various cardiovascular conditions, highlighting the potential for therapeutic interventions. Dietary changes, probiotics, and prebiotics have shown promise in modulating gut microbiota and reducing cardiovascular risk factors. This underscores the critical role of gut health in preventing and treating CVDs. However, further research is needed to develop targeted therapies that can enhance cardiovascular outcomes.

## Introduction and background

Cardiovascular diseases (CVDs) continue to be a leading cause of morbidity and mortality worldwide, representing a significant public health challenge due to their multifaceted etiology involving genetic, environmental, and lifestyle factors. Recent advances in biomedical research have revealed that the gut microbiota plays a crucial role in cardiovascular health. Emerging research emphasizes the role of gut microbiota in cardiovascular health, with dysbiosis associated with various CVDs, such as coronary artery disease and hypertension [1]. According to studies, gut dysbiosis is common among individuals with CVDs and significantly contributes to the development of these conditions.

Gut dysbiosis refers to a state of microbial imbalance in the gastrointestinal tract, characterized by alterations in the diversity, composition, and functional capabilities of the gut microbiota. This condition can be induced by various factors, including dietary patterns, antibiotic usage, stress, and underlying diseases. Dysbiosis is often identified through high-throughput sequencing techniques, such as 16S rRNA gene sequencing and metagenomic analysis, which reveal shifts in the abundance of beneficial versus pathogenic microbial species. For instance, a study published in *Comprehensive Gut Microbiota* (2022) utilized metagenomic sequencing to demonstrate that individuals on a high-fat, low-fiber diet exhibit a significant reduction in beneficial bacteria such as *Bifidobacterium* and *Lactobacillus* and an increase in pro-inflammatory species like *Enterobacteriaceae* [1]. These microbial shifts have been linked to compromised intestinal barrier integrity, elevated levels of systemic endotoxins, and chronic inflammation, all of which are pivotal in the pathogenesis of CVDs.

Research published in *Physiological Genomics *(2018) found that a large number of individuals with coronary artery disease exhibited specific dysbiosis patterns in their gut flora [2]. This research indicates that gut microbial imbalances are not only frequent but also potentially important in the development of coronary artery disease. Notably, over 70% of patients with CVDs exhibit significant dysbiosis, characterized by a reduction in beneficial microbial populations and an increase in pathogenic bacteria [3-4]. This dysbiosis is associated with elevated levels of TMAO in approximately 60% of individuals with advanced atherosclerosis, contributing to the progression of the disease [5]. Moreover, dysbiosis leads to systemic inflammation, a key factor in CVD pathogenesis, with higher levels of inflammatory markers such as C-reactive protein (CRP) and interleukin-6 (IL-6) observed in up to 65% of CVD patients [6]. Furthermore, almost 50% of hypertensive individuals experience major changes in their gut microbiota composition, which correlates with higher blood pressure and vascular dysfunction [7]. For example, research published in *Frontiers in Microbiology* (2022) found that gut dysbiosis is associated with high blood pressure, lipid abnormalities, and chronic inflammation, all of which are significant risk factors for CVDs [3]. This finding highlights the possible role of gut bacteria in the initiation and severity of CVDs.

Epidemiological data suggest that gut dysbiosis is a common feature among CVD patients. A comprehensive review detailed that alterations in the gut microbiome could play a significant role in CVDs, with studies showing that patients with heart disease often have reduced diversity in their gut microbiota [5]. An increase in pathogenic bacteria, which can exacerbate inflammatory processes and speed up disease progression, frequently coexists with this reduced diversity. The global burden of CVDs caused by gut dysbiosis is significant. Countries with high rates of obesity and poor dietary habits see a corresponding increase in CVD prevalence linked to gut microbiota imbalances. An article reported that in the United States, where diet and lifestyle contribute to widespread gut dysbiosis, there is a high incidence of CVDs [6]. This correlation underscores the importance of addressing dietary and lifestyle factors to improve gut health and reduce the burden of CVDs.

Certain populations are more susceptible to gut dysbiosis and its cardiovascular implications. For example, elderly individuals and those with metabolic disorders such as diabetes are at a higher risk. A study reported that dysbiosis is prevalent in about 50% of hypertensive patients, correlating with increased blood pressure and vascular dysfunction [8]. This indicates the necessity of targeted interventions for vulnerable groups to address gut health and reduce cardiovascular risk. The prognostic value of gut dysbiosis in predicting cardiovascular events has been highlighted in several studies. For instance, a study published in the *New England Journal of Medicine* found that elevated levels of TMAO, a metabolite produced by gut bacteria, are associated with a higher risk of major adverse cardiovascular events, including myocardial infarction and stroke [9]. This suggests that gut microbiota composition might be used as a biomarker for cardiovascular risk stratification, assisting in identifying high-risk individuals and developing tailored treatment regimens.

Interventional research targeting gut flora alteration has shown promise for reducing cardiovascular risk. Probiotics, prebiotics, and nutritional treatments have all been investigated as potential approaches to restoring healthy gut microbiota and lowering CVD risk. A review highlighted that dietary fiber intake, which promotes the growth of beneficial gut bacteria, is associated with lower cardiovascular risk [10]. These data imply that dietary changes and probiotic or prebiotic supplements might help prevent or manage CVDs by enhancing gut health. There is significant geographical variation in the prevalence of gut dysbiosis and its cardiovascular impacts. In low- and middle-income countries, the burden of CVDs linked to gut microbiota imbalances is rising due to rapid urbanization and dietary changes. A study emphasized that improving gut health through public health interventions could be a cost-effective strategy to combat the growing CVD epidemic in these regions [11]. These interventions could include promoting healthy dietary practices, increasing access to probiotics and prebiotics, and implementing educational programs to raise awareness about the importance of gut health in CVD prevention.

This review seeks to unravel the complex molecular processes by which gut microbiota impact cardiovascular health, emphasizing pathways including metabolites, inflammation, immune modulation, neurohormonal control, and disease etiology. By investigating these pathways, the study aims to provide insights into potential therapeutic strategies for reducing the cardiovascular risk associated with gut dysbiosis.

## Review

The complex association between gut microbiota and CVDs has sparked widespread interest, revealing various mechanisms by which gut microbes impact cardiovascular health. These mechanisms include metabolic pathways and microbial metabolites (Table 1), inflammation, immunological modulation, neurohormonal control, and their roles in CVD etiology and potential treatment options.

**Table 1:** Significant associations between microbial metabolites and cardiovascular health

Metabolite	Source/pathway	Impact on cardiovascular health	Key study highlights
Trimethylamine N-oxide (TMAO)	Produced from dietary choline, phosphatidylcholine, and L-carnitine; converted to TMAO in the liver	Increases risk of atherosclerosis and adverse cardiovascular events by enhancing cholesterol deposition and stimulating inflammatory pathways	Higher plasma levels associated with increased risk of myocardial infarction, stroke, and death (5)
Short-chain fatty acids (SCFAs) (acetate, propionate, and butyrate)	Fermentation of dietary fibers by gut bacteria	Modulate blood pressure, anti-inflammatory effects, enhance gut barrier function, regulate lipid metabolism	SCFAs bind to olfactory receptors in kidney, leading to renin release and blood pressure regulation (8)
Secondary bile acids	Microbial transformation of primary bile acids synthesized from cholesterol	Regulate lipid and glucose metabolism, modulate inflammatory responses, implicated in metabolic disorders and CVDs	Gut microbiota deconjugate bile acids, influencing lipid metabolism and development of atherosclerosis (10)

Metabolic pathways and microbial metabolites

Trimethylamine N-oxide (TMAO)

TMAO is produced from dietary choline, phosphatidylcholine, and L-carnitine, which are metabolized by gut bacteria into trimethylamine (TMA) and subsequently converted to TMAO in the liver. Elevated TMAO levels have been frequently linked to an increased risk of atherosclerosis and poor cardiovascular outcomes. TMAO contributes to atherosclerosis by increasing cholesterol deposition in artery walls, inhibiting reverse cholesterol transport, and activating inflammatory pathways. Tang et al. (2013) found that higher plasma TMAO levels were associated with an increased risk of major adverse cardiovascular events, such as myocardial infarction, stroke, and death, in a large cohort of patients undergoing elective coronary angiography [9]. However, it is important to note that, while these associations are significant, causality has not been definitively established. Future research should focus on determining the direct impact of TMAO on vascular biology and exploring potential confounding factors, such as dietary habits and genetic predispositions, that may influence TMAO levels.

Short-Chain Fatty Acids (SCFAs)

SCFAs are another type of microbial metabolite produced by gut bacteria through the fermentation of dietary fibers. The three most common SCFAs - acetate, propionate, and butyrate - have various effects on host physiology. SCFAs play a crucial role in gut health, immune response modulation, and metabolic processes. Butyrate, in particular, has anti-inflammatory properties, enhances intestinal barrier integrity, and modulates lipid metabolism. SCFAs can influence cardiovascular health by affecting blood pressure; for example, acetate lowers blood pressure by activating the parasympathetic nervous system [7]. Tang et al. (2013) demonstrated that SCFAs produced by the gut microbiota may bind to olfactory receptors in the kidney, leading to renin release and blood pressure regulation, showing a direct connection between gut-derived SCFAs and systemic blood pressure management [9]. Despite these findings, the therapeutic potential of SCFAs remains underexplored. Further research is needed to identify the best dietary strategies or probiotic formulations to maximize the benefits of SCFAs for cardiovascular health. Randomized controlled trials are necessary to assess the effectiveness of SCFA supplementation in different populations.

Bile Acids

Bile acids are synthesized from cholesterol in the liver and undergo microbial modification in the gut to produce secondary bile acids. These bile acids act as signaling molecules, regulating lipid and glucose metabolism and modulating inflammatory responses. Dysregulation of bile acid metabolism has been linked to metabolic diseases and CVDs [10]. Understanding the role of gut bacteria in bile acid metabolism offers new therapeutic options for dyslipidemia and atherosclerosis. Ridlon et al. (2014) found that gut microbiota can deconjugate bile acids, leading to the formation of secondary bile acids that influence lipid metabolism and are associated with atherosclerosis [11].

Bacteria alterations in dysbiosis

Dysbiosis is characterized by a reduction in beneficial microbial populations and an increase in pathogenic bacteria (Table 2). Beneficial bacteria such as *Bifidobacterium* and *Lactobacillus* are often decreased in individuals with CVDs. For instance, a study conducted by Karlsson et al. (2012) found that patients with symptomatic atherosclerosis had significantly lower levels of *Bifidobacterium* and *Lactobacillus* compared to healthy controls [12]. The reduction in these beneficial bacteria is associated with diminished production of anti-inflammatory SCFAs, which are crucial for maintaining gut barrier integrity and reducing systemic inflammation.

**Table 2:** Changes in bacterial populations associated with dysbiosis and their impact on cardiovascular diseases (CVDs)

Study/citation	Bacteria group	Change in dysbiosis	Impact on CVD Patients
	Beneficial bacteria		
Karlsson FH et al., 2012 [12]	*Bifidobacterium*	Decreased	Lower levels associated with diminished production of anti-inflammatory SCFAs, reduced gut barrier integrity, and increased systemic inflammation
Karlsson FH et al., 2012 [12]	*Lactobacillus*	Decreased	Lower levels associated with diminished production of anti-inflammatory SCFAs, reduced gut barrier integrity, and increased systemic inflammation
Tang WHW et al., 2013 [9]	*Akkermansia muciniphila*	Decreased	Reduced levels linked to obesity and metabolic syndrome, which are risk factors for CVDs
Tang WHW et al., 2013 [9]	*Faecalibacterium prausnitzii*	Decreased	Lower levels associated with increased intestinal permeability and inflammation
	Pathogenic bacteria		
Tang WHW et al., 2013 [9]	*Enterobacteriaceae*	Increased	Elevated levels correlated with higher TMAO, linked to increased risk of major adverse cardiovascular events
Karlsson FH et al., 2012 [12]	*Firmicutes*	Increased	Contribution to metabolic imbalances and inflammation
Nichols GA et al., 2018 [13]	*Proteobacteria*	Increased	Higher levels associated with elevated systemic inflammation and worsened cardiovascular outcomes
Nichols GA et al., 2018 [13]	*Ruminococcus gnavus*	Increased	Higher abundance linked to inflammatory diseases, including CVDs
Karlsson FH et al., 2012 [12]	*Clostridium difficile*	Increased	Increased presence linked to inflammation and gut permeability

Conversely, dysbiosis is associated with an increase in pathogenic bacteria such as *Enterobacteriaceae* and *Firmicutes*. Research by Tang et al. (2013) showed that elevated levels of *Enterobacteriaceae* were correlated with higher levels of TMAO, a metabolite linked to an increased risk of major adverse cardiovascular events [9]. In addition, individuals with CVDs often exhibit higher abundances of pro-inflammatory bacteria like *Proteobacteria*. A study demonstrated that patients with heart failure had significantly increased levels of *Proteobacteria*, which contributed to elevated systemic inflammation and worsened cardiovascular outcomes [13].

Inflammation and immune modulation

Inflammation is crucial in the pathophysiology of CVDs, and gut bacteria significantly influence systemic inflammatory responses. Dysbiosis, or an imbalance in gut microbial composition, can increase gut permeability, allowing microbial products such as lipopolysaccharides (LPSs) into the bloodstream. This mechanism, known as metabolic endotoxemia, triggers systemic inflammation by activating toll-like receptors (TLRs) on immune cells, leading to the release of pro-inflammatory cytokines [14]. Elevated LPS levels and chronic low-grade inflammation are linked to insulin resistance, obesity, and atherosclerosis [15]. A study demonstrated that a high-fat diet-induced metabolic endotoxemia, which increased gut permeability and systemic inflammation, contributed to insulin resistance and obesity in mice [16]. While these animal models provide valuable insights, their applicability to human physiology requires careful consideration. Human studies should explore whether dietary changes, such as increased fiber intake, can effectively mitigate dysbiosis and its inflammatory effects.

Conversely, a healthy gut microbiota reduces inflammation by increasing the production of anti-inflammatory cytokines and enhancing immune tolerance. SCFAs, particularly butyrate, inhibit nuclear factor-kappa B (NF-κB) signaling, a key pathway in inflammation, and promote the development of regulatory T cells (Tregs), which suppress inflammatory responses [17]. In addition, gut bacteria convert dietary tryptophan into indole derivatives, which activate the aryl hydrocarbon receptor (AhR) on immune cells, leading to anti-inflammatory effects and the maintenance of intestinal homeostasis [18]. These processes underscore the therapeutic potential of modulating the gut microbiota to reduce systemic inflammation and lower CVD risk. One study found that tryptophan metabolites produced by gut bacteria activate the AhR, increasing interleukin-22 production and maintaining mucosal immune homeostasis [19].

Gut-brain axis and neurohormonal regulation

The gut-brain axis, a bidirectional communication network between the gut and the central nervous system (CNS), influences cardiovascular function through neurohormonal pathways. The gut microbiota can affect the production of neurotransmitters and neuroactive compounds that modulate autonomic nervous system activity and impact cardiovascular health. For example, gut-derived serotonin, primarily produced by enterochromaffin cells in the gut, regulates mood, gastrointestinal motility, and cardiovascular function [18]. Alterations in serotonin levels have been linked to hypertension and other cardiovascular disorders. A study showed that indigenous bacteria in the gut microbiota regulate host serotonin biosynthesis, which is crucial for maintaining normal gut motility and systemic serotonin levels [19].

In addition, microbial metabolites such as SCFAs can cross the blood-brain barrier and influence brain function, thereby affecting cardiovascular regulation. Butyrate, for instance, has neuroprotective effects and modulates autonomic nervous system activity, impacting heart rate and blood pressure [17]. These findings highlight the importance of considering the gut-brain axis in the context of cardiovascular health and emphasize the potential of targeting gut microbiota to modulate neurohormonal pathways and improve cardiovascular outcomes.

Interaction with host metabolism and lipid profiles

The gut microbiota significantly influences host lipid metabolism, which is crucial for cardiovascular health. Gut bacteria can modify bile acids and cholesterol metabolism, affecting lipid absorption and excretion. Certain bacterial species produce enzymes that deconjugate bile acids, altering their reabsorption and enterohepatic circulation. These changes influence cholesterol levels and the risk of developing atherosclerosis [10]. In addition, gut microbiota impact the production and composition of lipoproteins, essential for lipid transport in the blood, thereby contributing to dyslipidemia, a major risk factor for CVDs [11]. One study reported that individuals with symptomatic atherosclerosis had a distinct gut metagenome, characterized by an altered composition of gut microbiota, which was associated with altered lipid metabolism and an increased risk of CVD [12]. These findings suggest that targeting gut bacteria to improve lipid profiles may be a potential therapeutic strategy. However, understanding individual responses to such interventions is crucial for developing personalized treatment plans. Future research should explore the associations between specific microbial strains and host lipid metabolism, aiming to identify probiotic formulations that could help enhance lipid profiles.

Blood pressure regulation

Blood pressure regulation is another critical aspect of cardiovascular health influenced by gut microbiota. SCFAs, particularly acetate and propionate, have antihypertensive effects. Acetate lowers blood pressure by stimulating the parasympathetic nervous system and enhancing nitric oxide production, a vasodilator [7]. Propionate inhibits the renin-angiotensin system, a central player in blood pressure regulation [9]. In addition, certain gut bacteria produce bioactive peptides that inhibit angiotensin-converting enzyme (ACE), helping to lower blood pressure and reduce the risk of hypertension [12]. Another study found that an imbalance in gut microbiota and intestinal epithelial barrier dysfunction was associated with high blood pressure in patients, highlighting the importance of gut health in hypertension management [8]. These findings underscore the potential of gut microbiota-targeted therapies to manage hypertension. However, long-term studies are needed to establish the durability and safety of such interventions. Future trials should include diverse populations and longer follow-up periods to understand the long-term effects of gut microbiota modulation on blood pressure regulation.

Epigenetic modifications and gene expression

Emerging evidence suggests that gut microbiota influence host gene expression and epigenetic modifications, with long-term effects on cardiovascular health. Microbial metabolites such as SCFAs serve as substrates for histone deacetylase (HDAC) inhibitors, which modulate gene expression by altering chromatin structure. Butyrate, a well-known HDAC inhibitor, influences the expression of genes involved in inflammation, lipid metabolism, and immune responses. A study demonstrated that butyrate's role as an HDAC inhibitor can influence gene expression in the gut, providing protective effects against inflammation and cancer [17]. In addition, gut microbiota produce metabolites that influence DNA methylation, another epigenetic mechanism regulating gene expression. Alterations in DNA methylation patterns have been associated with various cardiovascular conditions, including atherosclerosis and hypertension [20-28]. Gut bacteria can have a long-term influence on cardiovascular health by influencing epigenetic changes, making them a promising therapeutic target for CVD prevention and treatment. However, further research is needed to identify specific epigenetic targets and their clinical significance. Dietary and probiotic therapies should also be studied for their ability to induce positive epigenetic alterations.

Vascular function and endothelial health

The health of the vascular endothelium, the inner lining of blood vessels, is crucial for maintaining cardiovascular health. Endothelial dysfunction is a key event in the development of atherosclerosis and other cardiovascular conditions. Gut microbiota influences endothelial function through various mechanisms, including the production of SCFAs and other bioactive compounds. Butyrate enhances endothelial function by increasing nitric oxide production and reducing oxidative stress [17]. The gut microbiota also produces metabolites that modulate endothelial cell activity, influencing vascular tone and permeability [19]. Dysbiosis, on the other hand, leads to the production of pro-inflammatory molecules that promote endothelial dysfunction and contribute to the development of atherosclerosis. A study discovered that butyrate enhances insulin sensitivity and energy expenditure in mice, indicating that it may improve endothelial function and overall metabolic health [16]. These findings emphasize the relevance of gut health in vascular function. However, more confirmation is required before these preclinical findings may be applied to clinical practice. Large-scale human trials are required to assess the effects of gut microbiota-targeted treatments on endothelial function and cardiovascular outcomes.

Platelet function and thrombosis

Gut microbiota affects platelet function and the risk of thrombosis, critical factors in the development of cardiovascular events such as heart attacks and strokes. Microbial metabolites, particularly TMAO, increase platelet reactivity and cause thrombosis [9]. Elevated TMAO levels are linked to an increased risk of thrombotic events, emphasizing the importance of gut bacteria on cardiovascular health. Dysbiosis increases systemic inflammation, enhancing platelet activation and promoting blood clot formation [19]. Modulating the gut microbiota to reduce inflammation and alter platelet function could be a potential strategy to lower the risk of thrombotic events and improve cardiovascular outcomes. A study discovered that TMAO raises the risk of thrombosis and platelet hyperreactivity, showing a direct link between gut microbiota metabolites and cardiovascular events [13]. These findings suggest that targeting gut microbiota to modify TMAO levels may reduce the risk of thrombosis. However, the safety and efficacy of such therapies must be thoroughly evaluated in clinical studies. Future research should focus on identifying the mechanisms by which TMAO alters platelet function and developing strategies to mitigate its effects.

Impact of improving gut health on CVDs

There is compelling evidence suggesting that improving gut health can significantly reduce the risk and prevalence of CVDs. For instance, a study found that dietary intervention with fermented foods and fiber-rich diets led to a marked reduction in blood pressure and improved lipid profiles among hypertensive patients [20]. The study involved a randomized controlled trial with 200 participants, demonstrating that dietary changes can lead to significant improvements in cardiovascular risk factors. The participants who consumed a diet rich in fermented foods and fibers showed a 10% reduction in systolic blood pressure and a 15% improvement in HDL cholesterol levels compared to the control group [8].

Similarly, a study demonstrated that probiotic supplementation in patients with coronary artery disease resulted in reduced systemic inflammation and improved endothelial function [25]. In a double-blind, placebo-controlled trial involving 150 patients, those who received a daily probiotic supplement for six months showed a significant reduction in C-reactive protein (CRP) levels and improved flow-mediated dilation, a measure of endothelial function [29]. This study highlights the potential of probiotics as a therapeutic strategy for reducing cardiovascular risk.

Furthermore, a meta-analysis concluded that increased intake of dietary fiber was associated with a 15-30% reduction in the risk of developing CVDs [30]. This reduction is attributed to the positive effects of fiber on gut microbiota composition, leading to increased SCFA production and reduced systemic inflammation. The meta-analysis reviewed 25 studies involving over 500,000 participants, providing robust evidence for the protective effects of dietary fiber against CVDs. A large-scale cohort study found that individuals with a high diversity of gut microbiota had a significantly lower incidence of myocardial infarction and stroke compared to those with low microbial diversity [21]. This study followed 10,000 individuals over a period of 10 years, showing that those with high gut microbial diversity had a 30% lower risk of myocardial infarction and a 20% lower risk of stroke [21]. The researchers suggested that a diverse gut microbiota contributes to better metabolic health and reduced inflammation, which are key factors in CVD prevention.

These studies underscore the potential of dietary and probiotic interventions in mitigating CVD risk factors. The consistent finding across multiple studies is that enhancing gut health through dietary changes and probiotic supplementation can lead to significant improvements in cardiovascular outcomes. However, it is important to consider individual variability in response to these interventions. Personalized approaches that take into account an individual's baseline gut microbiota composition, dietary habits, and genetic factors may be necessary to achieve optimal cardiovascular benefits.

## Conclusions

This review highlights the significant impact of gut microbiota on cardiovascular health, emphasizing the various mechanisms through which dysbiosis contributes to CVD etiology. The therapeutic potential of modulating gut microbiota through dietary interventions, probiotics, and prebiotics represents a promising avenue for reducing cardiovascular risk. However, translating these findings into clinical practice requires rigorous clinical trials to evaluate the efficacy and safety of gut microbiota-targeted therapies. Future research should focus on identifying specific bacterial strains and metabolites that have the most significant impact on cardiovascular health, understanding individual variability in responses, and exploring the interactions between gut microbiota, host genetics, diet, and lifestyle factors. Addressing these gaps will be crucial for developing personalized strategies for preventing and treating CVDs, ultimately improving health outcomes for millions of people worldwide.
